# Assessing the effectiveness of a diabetes group visit training for health center staff: a pilot study of five Midwestern community health centers

**DOI:** 10.1186/s12913-022-08108-w

**Published:** 2022-06-04

**Authors:** Priscilla A. Barnes, Ivana Barouhas, Erin M. Staab, Amanda Benitez, Jefferine Li, Amanda Campbell, Cynthia T. Schaefer, Michael Quinn, Arshiya A. Baig

**Affiliations:** 1 Department of Applied Health Science, Indiana University School of Public Health, 1025 E. 7th St., Suite 296E, Bloomington, IN 47405 USA; 2 Pritzker School of Medicine, University of Chicago, Chicago, USA; 3 Section of General Internal Medicine, Department of Medicine, University of Chicago, Chicago, IL USA; 4 Enlace Chicago, Chicago, IL USA; 5 MidWest Clinicians’ Network, Inc., 321 W. Lake Lansing Road, East Lansing, MI 48823 USA

**Keywords:** Diabetes group visits, Community health centers, Federal qualified health center staff training, Perceived assets and obstacles, Formative evaluation research

## Abstract

**Background:**

Diabetes group visits are shared appointments that include diabetes education in a group setting and individual visits with a medical provider. An 18-month pilot study was designed to evaluate organizational capacity and staff preparedness in implementing and sustaining diabetes group visits.

**Results:**

Data were collected and analyzed from pre-post assessments and key informant interviews with community health center (CHC) staff (*N* = 26) from teams across five Midwestern states. Overall, participants demonstrated high baseline knowledge and awareness about diabetes group visit implementation. Changes in attitudes and practices did occur pertaining to familiarity with billing and increased awareness about potential barriers to diabetes group visit implementation. Key assets to diabetes group visit implementation were access to pre-designed resources and materials, a highly motivated team, and supportive leadership. Key obstacles were socioeconomic challenges experienced by patients, constraints on staff time dedicated to group visit implementation, and staff turnover.

**Conclusions:**

Results of the study provide a framework for implementation of diabetes group visit trainings for CHC staff. Future research is needed to assess the training program in a larger sample of CHCs.

In the U.S., approximately 34.2 million adults have diabetes [1]. Individuals with diabetes are highly susceptible to major complications such as renal failure, coronary artery disease, amputations, and diabetic retinopathy [2, 3]. In 2017, the economic cost of diagnosed diabetes was $327 billion, an increase from $245 billion in 2012 [4]. Approximately 60% was attributed to hospital inpatient care and prescribed medications to treat complications [4].

Diabetes group visits are becoming a popular practice for use in health care settings to improve patient outcomes. Diabetes group visits typically entail an individual consultation and physical exam combined with an in-depth group learning session [5, 6]. Diabetes group visits vary in structure and participation. The size of diabetes group visits can range from as small as 8 to as large as 20 patients each session [7]. The overall benefit of diabetes group visits for individuals living with diabetes is improvement in glycated hemoglobin [8, 9] and improved cardiovascular benefits following group visit discharge [10]. Clinics implementing diabetes group visits have also observed reduction in medical costs and decreased emergency department visits and hospitalizations. For patients, a major benefit of diabetes group visits is a dedicated space for health care professionals to provide diabetes self-management education outside the time-constrained setting of one-on-one medical visits [6, 10–14]. These visits have been noted to provide a social support network for patients as well as improve patient-provider communication [12–14].

Prior studies have examined key factors surrounding the implementation of diabetes group visits in different clinical settings such as Veteran Affairs hospitals and primary care settings [7–9, 11–13, 15, 16]. There are limited research studies in community health centers (CHCs), especially related to the adoption and implementation of diabetes group visits [17–19]. These health centers [20] play a vital role in caring for patients with diabetes; type 2 diabetes was diagnosed at a rate of 5.7% in patients ages 18–64 at CHCs, compared to 1.6% in physician offices [20–22].

The purpose of this study was to test a pilot educational intervention designed to prepare health center staff to implement diabetes group visits in five Midwestern CHCs. The central hypothesis was that a professional development training intervention would improve staff’s preparedness to implement and sustain diabetes group visits at their sites. Focusing on first-time implementation of diabetes group visits in CHCs presents opportunities to identify personnel, infrastructure and resources needs, especially in providing care for medically underserved populations.

## Methods

### Participant recruitment

Community health centers (CHCs) were recruited via e-mails sent to the Midwest Clinicians’ Network (MWCN) [23], a nonprofit organization that coordinates networking, educational opportunities, and research for its members in ten Midwestern states. The MWCN Research Committee and investigators at the University of Chicago selected six CHCs from nine that completed an application in response to recruitment emails. Sites selected for the diabetes group visit study were required to be community health centers, be members of MWCN, and have three staff members or volunteers 18 years of age or older who were available to participate. They were also required to have the support of the health center’s executive director/chief executive officer and medical director in enrolling in the study.

The selected CHCs included seven sites from six health centers located in Illinois, Indiana, Michigan, Nebraska, and Ohio. One CHC elected to participate at two sites, bringing the total to seven sites. Each site chose three to four employees to act as group visit trainees in the study, yielding 26 initial participants. One participant at each site acted as team leader for the group visit program and each team included a medical provider. The number of adult patients with type 2 diabetes at these sites ranged from 845 to 2450. All methods were performed in accordance with the research and scientific guidelines and regulations approved by the University of Chicago Institutional Review Board (Protocol # IRB 12–1839). Written informed consent was obtained from participants prior to the start of the study.

### Study design

**Fig. 1:**
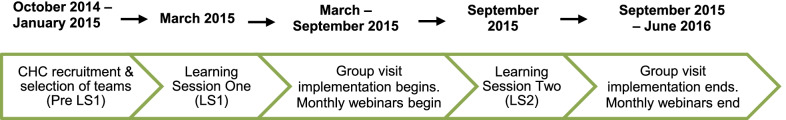
Training timeline

The 18-month training included a 2-day in-person learning session in Chicago, IL in March 2015 (Learning Session 1 [LS1]), a similar session in September 2015 (LS2), and monthly webinars to prepare health center staff to implement the group visit intervention at their CHCs (Fig. 1). Training modules were designed based on an extensive review of the diabetes research literature, data collected from site visits conducted with CHCs that had experience with diabetes group visits, and the guidance of the MWCN Research Committee. Topics included diabetes group visits model and structure, patient recruitment, principles of behavior change, strategies for gaining staff buy in, and discussion about overcoming potential barriers and sustaining diabetes group visits. Training was led by the study team, which included a physician, a nurse investigator, a behavioral scientist, the executive director of MWCN, project managers, and research assistants. Guest speakers were also invited based on their expertise on certain topics, such as patient recruitment and retention or experience with running group visits at a CHC. Staff received a group visit guide including planning worksheets and publicly available toolkits, curricula, and patient education materials.

After LS1, staff returned to their respective CHCs to carry out six monthly diabetes group visits. Group visits generally consisted of a patient check-in portion in which staff measured vital signs, a group activity, and a brief one-on-one provider visit for each patient. Each group visit session typically had a theme, such as healthy diet. Two CHCs offered bilingual sessions. During monthly webinars following LS1, participants reported on the challenges and successes they had as they prepared for and implemented the group visits. The training staff and participants from other CHC sites then provided feedback aimed at helping the group visit trainees overcome obstacles. Monthly webinars also included additional presentations on topics to assist in group visit implementation, including: billing, patient selection and recruitment, consenting eligible patients, administering patient baseline surveys, baseline chart abstraction, “Plan Do Study Act” (PDSA) cycles of quality improvement, and further advice for launching group visits building off of the LS1 modules. At LS2, staff shared their progress to date and their respective site’s goals regarding group visits after the study’s conclusion.

### Data collection

Prior to training, study participants (*n* = 26) completed an online pre-training survey via REDCap, a secure web-based data collection and management application. This survey contained questions about participant demographics, experience with patient education and group visits, self-reported preparedness to conduct group visits, perceived CHC capacity to implement diabetes group visits, and perceived benefits and barriers to group visit implementation. At the end of LS 1 & 2, participants completed a paper survey. Questions from the pre-training online survey were repeated on the paper surveys to assess change in knowledge and ability in implementing diabetes group visits. Questions related to general beliefs about diabetes group visits were assessed on a five-point Likert scale with responses ranging from 1 = strongly disagree to 5 = strongly agree. In addition, participants assessed the degree to which specific barriers impeded implementation of diabetes group visits. These factors were assessed on a four-point Likert scale ranging from 1 = major barrier to 4 = not a barrier.

Two rounds of semi-structured telephone interviews with participants were also conducted. All group visit trainees were invited via email to complete the first round of interviews, which occurred in late July and August 2015, shortly after launching the group visits. In the second round of interviews, 12 trainees were invited (one team leader and one team member at each site after one site withdrew from the study). The second round was completed in November and December 2016, after completion of the group visits at all sites. All interviews were conducted by members of the study team, audio-recorded, and transcribed by a certified transcription company. Interviews were 30–60 minutes. Participants were asked about their experiences going through training, preparing for the group visit program, recruiting patients, and launching the program, with an emphasis on group visit barriers and factors for success.

### Data analysis

Twenty-one indicators were analyzed in SPSS 26.0: general beliefs about individual readiness and organizational capacity to deliver diabetes group visits, perceived barriers, and perceived benefits. Descriptive statistics were used to determine characteristics of the CHC staff at the start of the study. One-way Repeated Measures ANOVA was conducted to assess if there were significant changes in participants’ perception of each measure (e.g., barriers to implementation) across three time points, Pre-LS1, Post-LS1, and Post-LS2. In addition, paired samples t-tests were conducted to assess if there were significant changes in participants’ perceptions of one measure (billing for group visits) between two time points, Post-LS1 and Post-LS2. The number of observations (N) reflect the number of complete cases used in the analyses. *P* < 0.05 was considered significant.

Of 26 champions, 13 participants completed the first telephone interview, and seven participants completed the second interview. All seven of the CHC staff members who completed the second round had also completed the first round of interviews. Seven of the 13 participants who completed first-round interviews were leaders of the group visit team at their respective sites. Recordings were transcribed and coded using MS Excel. Two levels of analysis were used to analyze data. In the first level of analysis, open coding allowed the research team to segment single words, phases, or paragraphs extracted directly from the transcripts [24]. In the second level of analysis, categorical coding was used to assign each open code a distinct attribute or characteristic related to the concept under study [24]. Participants’ insights regarding the training sessions as preparation and ongoing implementation were noted. Four members of the study team coded transcripts and discussed the findings. The most reported perceived assets and obstacles in implementing and sustaining diabetes group visits were identified based on 85% agreement with codes by the study team.

## Results

Table 1 summarizes the participants’ prior experience and the healthcare roles they served at their respective CHCs. Most participants (85%) were non-Hispanic White females. While they had been in practice an average of 11.9 years, only 11.5% of respondents reported having prior experience conducting group visits. The majority (76.9%) had prior training in lifestyle coaching and motivational interviewing techniques.

**Table 1:** Group visit champion characteristics and experiences with patient care and group visits (*N* = 26)

Characteristic	N (%) or Mean (SD)
Age at Learning Session 1 (Mean ± SD)	44.0 ± 8.5
Female, N (%)	22 (85%)
Race/Ethnicity, N (%)	
Non-Hispanic White	22 (84.6%)
Non-Hispanic Black or African American	2 (7.7%)
Non-Hispanic Asian	1 (3.8%)
Hispanic, Latino, or Spanish origin	1 (3.8%)
Current positions at health centers, N (%)	
Registered Nurse	8 (30.8%)
Physician	4 (15.4%)
Administrator	3 (11.5%)
Dietitian	2 (7.7%)
Licensed Practical Nurse	2 (7.7%)
Nurse Practitioner / Advanced Practice Nurse	2 (7.7%)
Physician’s Assistant	2 (7.7%)
Health Educator	1 (3.8%)
Medical Assistant	1 (3.8%)
Social Worker	1 (3.8%)
Years practicing since completing training (Mean ± SD)	11.9 ± 10.0
Years working at current health center (Mean ± SD)	6.6 ± 6.4
Percentage with prior training in lifestyle coaching or motivational interviewing techniques, N (%)	20 (76.9%)
Percentage with prior experience conducting group visits, N (%)	3 (11.5%)
Health conditions covered in prior group visits, N (%)	Diabetes, 1 (3.8%)
	Obesity/Overweight, 1 (3.8%)
	Health Literacy, 1 (3.8%)

In general, the training increased participants’ awareness about what is needed for successful implementation of group visits at their health center. General awareness increased from 3.00 (SE = 0.24) prior to training, 4.38 (SE = 0.13) post-LS1, to 4.50 (SE = 0.13) post-LS2 [*p* = 0.023]. In addition, familiarity with billing for group visits increased from 2.94 post-LS1 to 3.59 post-LS2 [diff (SE) = 0.65 (0.26), *p* = 0.023] (Table 2). Overall, participants demonstrated high baseline knowledge of and motivation for implementing diabetes group visits. Although not statistically significant, participants believed their CHCs were prepared to conduct and continue diabetes group visits upon completion of the training. Moreover, participants felt that primary care providers in their CHCs would refer patients to diabetes group visits (Table 2).

**Table 2:** Differences in general beliefs related to diabetes group visit implementation pre- and post-learning sessions

General beliefs about diabetes group visits^a^		Pre-LS1	Post-LS1	Post-LS2	***p***-value
	N^b^	Mean (SE)	Mean (SE)	Mean (SE)	
My CHC is motivated to (*conduct/continue*) diabetes group visits at our center.	16	4.44 (0.23)	4.50 (0.13)	4.13 (0.22)	0.298
My CHC can (*get/has involved*) staff/providers involved in conducting diabetes group visits at our center.	16	4.19 (0.25)	4.44 (0.13)	4.00 (0.26)	0.233
My CHC believes that conducting diabetes group visits at our center will benefit our community.	16	4.44 (0.23)	4.50 (0.13)	4.19 (0.21)	0.414
My CHC can keep track of the progress in implementing diabetes group visits at our center.	16	4.31 (0.25)	4.31 (0.12)	4.06 (0.21)	0.498
My CHC has the resources needed to conduct diabetes group visits at our center.	16	4.13 (0.24)	4.25 (0.14)	4.13 (0.22)	0.878
My CHC is prepared to (*conduct/continue*) diabetes group visits at our center.	16	3.88 (0.18)	4.19 (0.10)	4.31 (0.15)	0.146
My CHC (*will be able/has the ability*) to recruit sufficient numbers of patients with diabetes to attend our group visits.	17	4.18 (0.18)	4.18 (0.13)	3.94 (0.14)	0.379
Physicians at my CHC will (*refer/continue to refer*) patients to our diabetes group visits.	17	4.11 (0.17)	4.06 (0.16)	4.12 (0.17)	0.939
My team has the ability to track the group visit patient’s progress and health-related targets.	17	4.29 (0.11)	4.24 (0.14)	4.29 (0.17)	0.910
I am aware of what is needed to successfully implement group visits in a health center.	16	3.00 (0.24)	4.38 (0.13)	4.50 (0.13)	< 0.001*
I am prepared to address (*potential/future potential*) barriers to implementing and (*sustaining/continuing*) diabetes group visits.	17	4.12 (0.15)	4.41 (0.15)	4.29 (0. 14)	0.180
My team will be able to continue the diabetes group visit program for a year or more.	16	4.13 (0.09)	4.31 (0.12)	4.13 (0.18)	0.455
We are familiar with how to bill for group visits.	17	**Post-LS1**	**Post-LS2**	**Diff/SE**	0.023*
		2.94	3.59	0.65/0.26	

*p<0.05

^a^Statements were rated on a five-point Likert scale (1 -strongly disagree to 5-strongly agree)

^b^Total number of complete cases across time points

As for perceived benefits, participants were aware of the benefits of the group visit model of care prior to beginning the training; however, awareness of these benefits significantly increased throughout the course of the training (Table 3). General awareness increased from 4.00 (SE = 0.17) prior to training, 4.60 (SE = 0.13) post-LS1, to 4.60 (SE = 0.13) post-LS2 [*p* = 0.001]. More specifically, diabetes group visits were perceived as improving efficiency in patient care (4.06 (SE = 0.18) pre-LS1, 4.59 (SE = 0.12) post-LS1, to 4.53 (SE = 0.15) post-LS2 [*p* = 0.033]). An additional benefit noted by participants was that group visits can increase patient satisfaction with diabetes care (4.18 (SE = 0.13) pre-LS1, 4.53 (SE =0.13) post-LS1, to 4.53 (SE =0.15) post-LS2 [*p* = 0.045]). It is important to note that participants did not agree or disagree that group visits improved provider productivity (3.94 (SE = 0.18) pre-LS1, 3.71 (SE =0.17) post-LS1, to 3.18 (SE =0.21) post-LS2 [*p* = 0.013]) (Table 3).

**Table 3:** Differences in beliefs about perceived benefits of diabetes group visit implementation pre- and post-learning sessions

Statements related to perceived benefits^a^	N^b^	Pre-LS1	Post-LS1	Post-LS2	***p***-value
		Mean (SE)	Mean (SE)	Mean (SE)	
**I am aware of the benefits of the group visit model of care.**	15	4.00 (0.17)	4.60 (0.13)	4.60 (0.13)	0.001*
**Innovative programs, like group visits, make health centers more competitive in attracting patients.**	17	4.06 (0.18)	4.47 (0.15)	4.35 (0.15)	0.128
**Group visits have the potential to improve**					
patient outreach in the community	17	4.29 (0.14)	4.47 (0.15)	4.53 (0.13)	0.242
a health center’s standing in the community	17	4.29 (0.11)	4.53 (0.13)	4.53 (0.13)	0.171
efficiency in patient care	17	4.06 (0.18)	4.59 (0.12)	4.53 (0.15)	0.033*
the use of health CHC resources	17	4.35 (0.12)	4.59 (0.12)	4.41 (0.17)	0.244
**Group visits can help:**					
providers get to know their patients in a deeper manner	17	4.29 (0.14)	4.53 (0.13)	4.47 (0.15)	0.316
improve provider productivity	17	3.94 (0.18)	3.71 (0.17)	3.18 (0.21)	0.013*
improve coordination of care for patients	17	4.47 (0.15)	4.47 (0.13)	4.29 (0.19)	0.445
**Group visits can:**					
increase patient confidence and self-efficacy in diabetes management	17	4.41 (0.12)	4.59 (0.12)	4.53 (0.13)	0.379
boost CHC provider and staff morale	17	4.00 (0.15)	4.24 (0.16)	4.18 (0.20)	0.479
foster multidisciplinary collaboration amongst staff and providers	17	4.47 (0.13)	4.59 (0.12)	4.53 (0.15)	0.665
increase patient satisfaction with diabetes care	17	4.18 (0.13)	4.53 (0.13)	4.53 (0.15)	0.045*

*p<0.05

^a^Statements were rated on a five-point Likert scale (1 -strongly disagree to 5-strongly agree)

^b^Total number of complete cases across time points

Participants became more aware of the barriers of the group visit model of care (Table 4). Awareness of potential implementation barriers increased from 3.75 (SE = 0.19) pre-LS1, to 4.31 (SE = 0.12) post LS1, to 4.88 post-LS2 (SE = 0.09) [*p* = 0.001] (Table 4). Participants reported creating and maintaining a billing mechanism (3.12 (SE = 0.17) pre-LS1, 2.59 (SE =0.15) post-LS1, to 2.82 (SE =0.15) post-LS2 [*p* = 0.013]) as a significant organizational barrier, particularly after attending the learning sessions. Concerns regarding lack of individual medical attention (3.00 (SE = 0.17) pre-LS1, 3.06 (SE =0.16) post-LS1, to 3.65 (SE =0.12) post-LS2 [*p* = 0.002]) and confidentiality and privacy (2.88 (SE = 0.22) pre-LS1, 2.81 (SE =0.14) post-LS1, to 3.50 (SE =0.13) post-LS2 [*p* = 0.012]) were seen as patient barriers when staff began training but less so post-LS2. It is important to note that participants reported recruiting at least 12–15 potential patients as somewhat of a barrier at pre-LS1 [3.13 (SE = 0.18] and post-LS1 [3.06 (SE = 0.17)]. Following post-LS2, recruitment was noted as a moderate barrier [2.31 (SE = 0.24)].

**Table 4:** Differences in beliefs about perceived barriers to diabetes group visit implementation pre- and post-learning sessions

Statements Related to Perceived Barriers	N^b^	Pre-LS1	Post-LS1	Post-LS2	***p***-value
		Mean (SE)	Mean (SE)	Mean (SE)	
**I am aware of the potential barriers to implementing group visits in a health center.**^a^	16	3.75 (0.19)	4.31 (0.12)	4.88 (0.09)	0.001*
**To what degree are the following barriers to implementing group visits at your CHC?**^**3**^					
Gaining strong leadership, provider, and staff support	17	3.47 (0.19)	3.47 (0.15)	3.41 (0.19)	0.961
Organizational encouragement of disease management programs	16	3.63 (0.16)	3.63 (0.13)	3.81 (0.14)	0.387
Lack of financial incentives or gifts	17	3.12 (0.15)	2.94 (0.14)	3.06 (0.18)	0.620
Creating and maintaining a billing mechanism	17	3.12 (0.17)	2.59 (0.15)	2.82 (0.15)	0.013*
Collecting data to assess patient outcomes	17	3.71 (0.11)	3.47 (0.15)	3.47 (0.17)	0.355
Advanced planning for adverse events and staff turnover	17	3.24 (0.16)	3.29 (0.17)	2.94 (0.16)	0.072
Recruiting at least 12–15 potential patients	16	3.13 (0.18)	3.06 (0.17)	2.31 (0.24)	0.004*
**To what degree are the following barriers to patients who may want to attend group visits at your site?**^c^					
Lack of transportation	17	2.18 (0.18)	2.11 (0.15)	2.53 (0.19)	0.076
Lack of time	17	2.53 (0.19)	2.24 (0.16)	2.47 (0.15)	0.121
Lack of financial incentives or gifts	17	2.53 (0.19)	2.47 (0.15)	2.88 (0.21)	0.232
Disinterest in the group setting	17	2.59 (0.17)	2.65 (0.17)	2.77 (0.22)	0.612
Concerns regarding lack of individual medical attention	17	3.00 (0.17)	3.06 (0.16)	3.65 (0.12)	0.002*
Concerns regarding confidentiality and privacy	17	2.88 (0.22)	2.81 (0.14)	3.50 (0.13)	0.012*

*p<0.05

^a^Statements were rated on a five-point Likert scale (1-strongly disagree to 5-strongly agree)

^b^Total number of complete cases across time points

^c^Statements were rated on a four-point Likert scale (1-major barrier, 2-moderate barrier, 3-somewhat of a barrier, 4-not a barrier)

### Perceived assets and obstacles

Analysis from the semi-structured interviews revealed the most reported assets and obstacles to the implementation of the group visit program among 5 Midwestern CHCs. One team was unable to overcome the challenge of patient recruitment and withdrew from the study in late June 2015. Assets and obstacles reported by at least 3 CHCs were considered the most influential factors to diabetes group visit implementation.

The most notable asset was having a pre-designed toolkit. Four of the five CHC teams expressed that the training materials were key to preparing them for group visit implementation. These resources included a group visit planning worksheet, planning checklist, links to how-to guides for group visit implementation, diabetes education curricula, and billing instructions. The training materials were viewed by most participants as a valuable tool that guided the operationalization of each visit and its purpose within the context of the CHC. One participant noted, “The materials were really great. We have been able to get a lot out of them. They gave us a real lot of ideas for how to kind of tweak things and make it work at our center.” Learning sessions also provided an environment to discuss the group visit care model, use of patient education.

Another major asset was having a highly motivated team. One participant stated, “Everyone was on board and had a very good understanding of expectations before we even started [the visit] that day.” Another participant said, in reference to the importance of team motivation as a facilitator, “All of the people involved in this particular initiative believe in it and are excited about it and really take ownership of it, and I think that is what has made it successful.” High motivation was connected to CHC teams’ ability to be agile in implementing diabetes group visits. All five CHC teams learned to leverage existing human resources within their clinic setting. Clinics invited guest speakers to present topics in an effort to share the workload.

A third reported asset to diabetes group visit implementation was the presence of leadership support. A participant reflecting on the positive effect of leadership support on the actualization of the group visits stated, “Our clinical director and the medical staff, clinical staff, everyone is very supportive of this. That is our environment here anyway. We support each other, especially if it meets our mission, which this does.*”*

Time constraints in incorporating the diabetes group visit model into CHCs were cited as the major obstacle by all teams. Three of five CHC teams expressed the burden of carrying out their regular job responsibilities outside of implementing the group visit model. As a result, the burden of both responsibilities limited the time CHC staff could devote to the study. Study participants addressed their time limitations by working more hours, enlisting the help of other health center staff, and prioritizing their responsibilities.

Socioeconomic challenges experienced by patients were noted as another major obstacle for four of five CHCs. Participants shared challenges in working with their patient population. Although group visits were seen as a necessary service, it did not negate the personal and environmental factors that interfere with attendance to medical appointments and consistent diabetes self-management practices. All CHCs cited lack of transportation as a major obstacle for their patients. Although health centers made the program financially accessible by waiving co-pays and making transportation accessible, it was not enough of an incentive.

A third major obstacle reported by participants was staff turnover. Three teams each lost one member to staff turnover.

The final obstacle experienced by CHCs was patient recruitment. The CHC that withdrew from the study struggled to identify enough patients meeting the study’s inclusion criteria of poorly controlled diabetes and they did not have enough staff to make recruitment phone calls. This site was also facing many changes and new obstacles outside the study, such as health center audits and electronic medical record transitions.

## Discussion

Overall, staff possessed high interest and knowledge about diabetes group visits. Access to a pre-designed resources and materials, a highly motivated team and supportive leadership were identified as key facilitators to early adoption of visits in their CHCs. Socioeconomic challenges experienced by patients, constraints on staff time dedicated to group visit implementation, and staff turnover, were obstacles to ongoing implementation.

In general, staff acknowledged that the CHC training serves as a collaborative ‘space’ for clinical, educational, and administrative staff to simultaneously work together to build an infrastructure that supports initial implementation of diabetes group visits. In this study, in-person learning sessions and monthly team updates on webinars provided peer support, encouragement, and networking during the process of taking on a demanding project. These sessions improved awareness of potential implementation barriers after discussions regarding the patient (client focused), provider, and health center (organizational) level challenges the staff members were likely to encounter. Of note, by the time CHC staff completed the post-LS2 surveys, all sites had initiated their six-monthly group visits, and thus had been able to directly observe the true role of the potential barriers listed in the survey, rather than making conjectures as they did prior to LS1. In-person learning sessions were designed to provide direction in navigating potential implementation barriers, but only LS2 included protected time for sites to lead discussions detailing their experiences, including how they overcame challenges. In a broad sense, this elucidates the role of camaraderie and peer support in the process of taking on a demanding project. More specifically, it demonstrates the importance of the exchange of advice and guidance between CHCs – institutions that by design share many traits in common – launching similar initiatives (in this case, group visit programs).

Similarly, increased awareness about the barriers of the group visit model improved after LS2, by which time participants attended monthly webinars and experienced the launch or completion of the group visit programs at their respective sites. The group check-in portions of the monthly webinars and the site-led reflection sessions at LS2 provided a support network for participants to share their own experiences with that of their peers at other sites, perhaps affirming observations they had already made regarding the benefits of group visits, but also allowing them to assess the program from new vantage points. In addition, it is conjectured from the change in perception from moderate to somewhat a barrier, specifically seen in confidentiality and privacy concerns as well as lack of individual medical attention, helped to improve administrative processes within CHCs.

The lack of change in the mean score of the remainder of the survey results may be attributed to a “ceiling effect”. The participants gave high pre-training scores to many measures of CHC capacity for group visits, team ability to handle the demands of group visits, and the perception of the specific benefits of group visits. This may have been a result of the site selection process, which led to enrollment of CHCs that had many of the resources necessary to start a group visit program and team members who valued the group visit model prior to training. The training, however, did impact CHC staff familiarity with billing diabetes group visits. This is an area that was new to participants and an unexplored opportunity of most CHCs.

The training raised awareness about obstacles encountered during the implementation process. Some participants adapted to these obstacles by expanding patient recruitment to provider panels other than that of the group visit provider; enlisting the help of additional health center staff and volunteers to make recruitment phone calls, check patients in, and measure vital signs; allowing one-on-one makeup sessions after the first group visit; offering transportation services; and using patient information cards to expedite preparation of the one-on-one component of the visit. In addition, participants credited our training materials with helping them initiate and sustain the group visits. As examples of resources, the training guided participants through the challenges of patient recruitment and staff time limitations. CHC teams were given telephone recruitment scripts, sample request letters for primary care physicians to approve the recruitment of their patients, and presentations on how to address staff time limitations. We did not, however, measure the use of these resources by staff who found themselves navigating the challenges these materials were meant to address.

Analysis elucidated the portions of the training that were most essential to the improvement of overall participant preparedness to conduct diabetes group visits as reflected by our survey results and qualitative analysis. Ongoing discussions about both anticipated and unforeseen challenges, with evidence-based advice for how to overcome them, were perhaps the most impactful of the educational tools we provided. The study team also believe the provision of detailed guidelines for group visit logistics and curricula were essential. The peer learning aspect of our training program also bolstered the success of each site, as participants were able to share solutions to common barriers. Finally, our support regarding the navigation of intra-CHC relationships (for example, in staff recruitment and retention and garnering leadership support) appeared to be one of the key components of our training intervention.

Success in diabetes self-management, as observed at the patient level in group visits, is not only predicated on information delivery about the disease. Peer support, especially among people with common lived experiences, has been identified as an important factor that facilitates intention to consider new attitudes or adopt new behaviors related to improved diabetes self-care [8, 25]. In addition, diabetes group visits serve as a supportive network for individuals to keep each other accountable on goals as well as celebrate short- and long-term successes. A similar dynamic can also occur at the organizational level through interactive training models for diabetes group visits. Several diabetes group visits models exist [14, 26, 27] (e.g., Cooperative Health Care Clinics [CHCCs], Drop In Group Medical Appointments [DIGMAs], American Diabetes Association’s [ADA] Adherence to Diabetes Guidelines), but it is unclear if the trainings of these models apply instructor or student centered curricula. This pilot study provides a framework for delivering interactive diabetes group trainings involving multiple CHCs that share common organizational and environmental challenges in addressing type 2 diabetes. In addition, this study points to the importance of creating experiential active trainings that empower staff to solve ‘real-time’ challenges. Future studies can explore the aspects of collaborative learning and problem solving using a co-learning collaborative approach that allows multiple CHCs to engage in the early implementation of diabetes group visit at one time as opposed to doing it on their own.

There were several study limitations. The inclusion of only Midwestern states may limit application of the findings to other geographic areas. Another potential source of bias was the study team’s selection of CHCs that were likely to be successful, possibly causing us to overestimate the value of the educational intervention. In addition, it is possible that the training intervention attracted CHCs that were motivated or already positioned to deliver diabetes group visits. With lack of a comparison group, it is difficult to assess whether the trainees’ strategies for overcoming barriers stemmed from our trainings. Also, the completion of the telephone interviews by only a portion of the participants may have introduced a selection bias. Moreover, the ability to determine the extent to which the improvements measured after LS2 could be attributed to the learning session itself was limited. The participants had also gained experience running the program and had likely gained confidence and knowledge by simple trial and error.

This is the first study to assess the effectiveness of a diabetes group visit training for CHCs and sets the stage for ongoing exploration of training models in this organizational setting. Of the health centers participating in this training intervention, six of seven started group visits and five of seven conducted monthly group visits within the study period. Interest in group visits has prompt expansion of this initiative to other health centers. Two additional cohorts of MWCN affiliated health centers have been trained as part of an ongoing cluster randomized controlled trial of diabetes group visits. The results of this diabetes group intervention have shown significant improvements in patients’ hemoglobin A1C compared to patients who received usual care at CHC sites [28].

Specifically, this study heightened awareness about the challenges related to staff turnover and billing. There are numerous potential approaches that might speculatively improve implementation and sustainability. These might include, for example, changes in administrative infrastructure such as changing billing systems or obtaining state and federal grants to support specific staff to conduct group visits. At the same time, definitive recommendations would likely be premature at this point. Instead, an important next step is to studies using dissemination and implementation models (i.e., Consolidated Framework for Implementation Research) to explore the feasibility and mechanisms through which the intervention can be integrated within CHCs. Future studies involve examining the application of dissemination and implementation models to evaluate strategies employed by multidisciplinary teams in these health centers to sustain group visits. Also, future investigations of CHCs outside the Midwest, as well as CHCs with fewer resources and less staff buy-in than those followed in our study, are expected to yield valuable additions to our findings and to the results of prior research.

## Data Availability

The datasets generated and/or analyzed during the current study are not publicly available manuscripts in progress but are available from Dr. Arshiya Baig, University of Chicago upon reasonable request.

## Abbreviations

CHC: Community health center
MWCN: Midwest Clinicians’ Network
LS: Learning session
